# Comparison of injectate spread following transverse vs. sagittal *in-plane* ultrasound-guided thoracic paravertebral block: a cadaveric study

**DOI:** 10.3389/fmed.2025.1667862

**Published:** 2025-11-12

**Authors:** Jiali Tang, Shuai Tang, Naili Wang, Bing Bai, Yuelun Zhang, Yangyang Zhang, Manjiao Ma, Yaodan Bi, Xinhua Shen, Di Zhang, Chao Ma, Yuguang Huang

**Affiliations:** 1 Department of Anesthesiology, Peking Union Medical College Hospital, Peking Union Medical College & Chinese Academy of Medical Sciences, Beijing, China; 2 Experimental Teaching Center, Institute of Basic Medical Sciences Chinese Academy of Medical Sciences, School of Basic Medicine Peking Union Medical College, Beijing, China; 3 Institute of Clinical Medicine, Peking Union Medical College Hospital, Peking Union Medical College & Chinese Academy of Medical Sciences, Beijing, China; 4 Department of Human Anatomy, Histology and Embryology, Neuroscience Center, Institute of Basic Medical Sciences Chinese Academy of Medical Sciences, School of Basic Medicine Peking Union Medical College, Beijing, China

**Keywords:** nerve block, ultrasonography, regional anesthesia, pain management, dye spread, thoracic paravertebral block

## Abstract

**Background:**

Thoracic paravertebral block (TPVB) is a clinically valuable regional anesthesia and analgesia technique for managing postoperative acute pain and certain chronic pain conditions. There are several approaches for ultrasound-guided TPVB. However, currently it is hard to provide an evidence-based recommendation on the choice between approaches. Comparisons of injectate distribution patterns among different approaches are limited. This observational cadaveric study compared dye distribution following TPVB using transverse *in-plane* (TI) and sagittal *in-plane* (SI) ultrasound-guidance.

**Methods:**

Ten paravertebral injections at the T6-7 were performed on five cadavers. Left side received injections with TI approach, and right side with SI approach. All injections consisted of 20 mL of 0.02% methylene blue. The cadavers were dissected to evaluate dye distribution. The ChAracteristics of Cadaver Training and sUrgical Studies (CACTUS) guideline was adhered to conduct and report this study.

**Results:**

All paravertebral injections resulted in dye staining in paravertebral space (PVS). On average, 3 [IQR (3, 3)] PVS segments were stained with TI approach, and 2 [IQR (1, 2)] with SI approach (*p* = 0.26). Median intercostal staining area was 55.1 [IQR (30.1, 76.0)] cm^2^ with TI and 38.3 [IQR (7.8, 82.6)] cm^2^ with SI approach (*p* = 0.50). Sympathetic chain staining was observed in 80% (TI) and 40% (SI) of cadavers (*p* = 0.50). Regardless of injection approach, (1) the cephalad and caudal dye distribution was 1 [(IQR (0, 2)] and 0 [IQR (0, 1)] segment separately (*p* = 0.04); (2) a significantly higher odds of PVS staining, and a significantly longer distance of intercostal space stained were observed at T6-7 (*p* < 0.001, *p* < 0.01) and T5-6 level (*p* = 0.001, *p* < 0.01); (3) a positive association was observed between the number of PVS segments stained and sympathetic chain staining (*p* = 0.003).

**Conclusions:**

Both TI and SI ultrasound-guided TPVB approaches reliably target PVS. A predominantly cephalad distribution was noted with two approaches. No significant differences were observed between two approaches regarding the number of PVS segments stained, intercostal spread area, and the percentage of sympathetic chain stained. This study adds knowledge to the spread pattern of TPVB.

## 1 Introduction

Thoracic paravertebral block (TPVB) is a clinically valuable regional analgesia technique, widely applied for managing pain following thoracic and abdominal surgeries, as well as certain chronic pain conditions (1–6). TPVB involves the targeted delivery of drug solution to the paravertebral space (PVS), blocking the branching thoracic spinal nerves and sympathetic nerve fibers to produce unilateral analgesia (7, 8). This approach offers potential benefits over systemic opioid analgesia by minimizing systemic side effects and avoiding the risks associated with neuraxial techniques (9–12).

There are multiple possible techniques when performing a TPVB, including landmark-based techniques using pressure measurement (9), electrostimulation guidance (13, 14), as well as live ultrasound guidance (15). The increasing use of ultrasound imaging has facilitated the development of various ultrasound-guided TPVB approaches (15). Ultrasound-guided TPVB can be performed using either transverse or sagittal approaches, depending on transducer orientation. The transverse approach requires a longer needle path, making it suitable for *in-plane* advancement with good visualization, but the needle points toward the epidural space, raises concerns about a greater risk of epidural spread. The sagittal approach involves a shorter, steeper trajectory, which makes *in-plane* needle tip visualization more difficult but facilitates *out-of-plane* injections. From a learning perspective, placing the probe about 2 cm lateral to the spinous process in the sagittal plane and using an *out-of-plane* technique is generally easier for beginners compared with the transverse *in-plane* technique. These techniques differ mainly in needle trajectory, visualization, and safety considerations (15). However, the difference of injectate distribution patterns between transverse and sagittal approaches is still unclear.

Currently, the choice of TPVB approach with ultrasound guidance mainly depends on the tradition of institution, and preference of performer, rather than the specific spread pattern. Actually, understanding the spread pattern of local anesthetic (LA) following a particular ultrasound-guided TPVB approach is essential. Knowledge of the spread pattern allows for informed selection of the injection site and prediction of the resulting sensory distribution, leading to improved analgesic efficacy. While several studies have investigated the injectate spread within the PVS and nearby anatomical structures, as well as the distribution of sensory block after a specific ultrasound-guided TPVB approach (15), direct comparisons between different approaches with respect to injectate distribution patterns remain limited. Therefore, this study utilized a fresh cadaver model to compare the contrast dye distribution following ultrasound-guided TPVB at T6-7 performed via transverse *in-plane* (TI) vs. sagittal *in-plane* (SI) approaches. These two approaches are commonly used. This observational anatomical study aimed to quantify differences in the number of PVS stained, distance of intercostal spread, and sympathetic chain stained. By providing a detailed anatomical comparison of these two common TPVB techniques, this study sought to inform clinical practice, guide technique selection, and ultimately contribute to improved patient outcomes.

## 2 Methods

The procedures used in this study adhere to the tenets of the Declaration of Helsinki. Anatomical bodies were obtained from Beijing Voluntary Body Donation Registration and Acceptance Station affiliated with Peking Union Medical College. The donors had signed an informed consent for body donation during their lifetime, which explicitly stated that the body would be used for medical education and research. The post-mortem interval before preservation was less than 2 days. We adhered to the ChAracteristics of Cadaver Training and sUrgical Studies (CACTUS) guideline to conduct and report this study (https://www.equator-network.org/reporting-guidelines/reporting-characteristics-of-cadaver-training-and-surgical-studies-the-cactus-guidelines/). CACTUS checklist can be found at online resource (Supplementary material). 5 fresh un-embalmed human cadavers were studied. Prior to the study, the cadavers were preserved in an environment at −4 °C. The duration of preservation was within 24 h. None of cadavers exhibited thoracic or dorsal deformities, traumatic lesions, or surgical scars. Bilateral ultrasound-guided paravertebral injections were conducted at T6-7 in each cadaver in prone position, while in our center this position is more frequently used during TPVB procedure. TI approach was utilized on left side, and SI approach on the right side. All injections were performed by a single experienced regional anesthesia specialist. Subsequently, a detailed layer-by-layer dissection of the paravertebral region from T1 to T12 was conducted by a dedicated anatomist assisted by two trained technicians.

### 2.1 Ultrasound-guided thoracic paravertebral injection

A Sonosite Edge ultrasound device with a curved transducer (5-2 MHz) (Sonosite, Bothell, Washington, USA) was used. The method for vertebral level identification was adapted from previously described techniques (16). Briefly, ultrasound transducer was placed parallel to and approximately 4 cm lateral to spinous processes at the subcostal margin in prone position. An ultrasound image revealed two to three block-like bony shadows. Then move transducer laterally. The bony shadow at the cephalad margin persisted, though it became flatter and more superficial. In contrast, the caudal shadow disappeared. The persistent cephalad shadow represented the short-axis image of the 12th rib. The transducer was moved cephalad to locate 6th rib and 6th thoracic vertebra.

**Figure 1:**
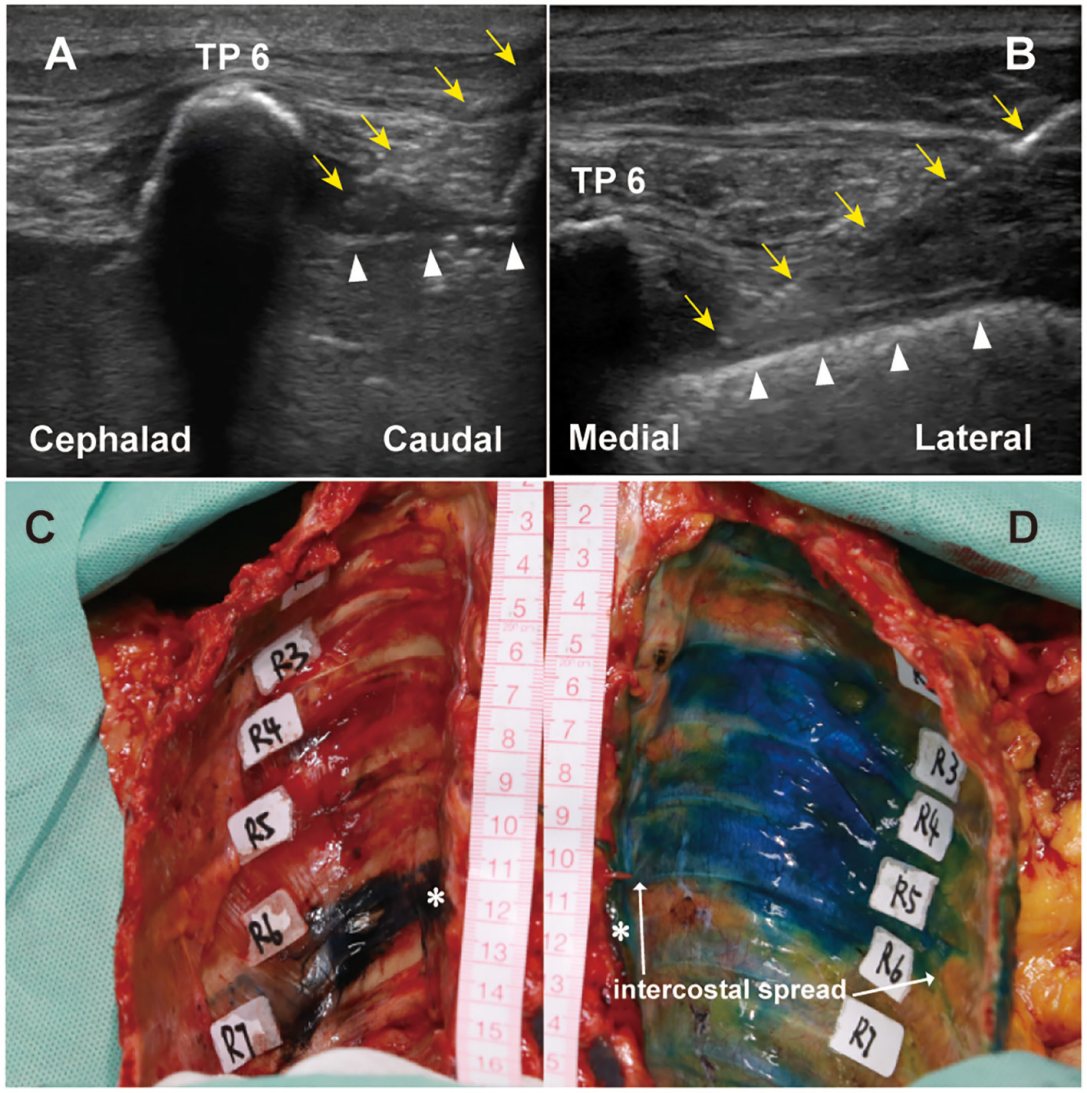
Ultrasound views of T6-7 paravertebral injections using two approaches and dye spread in PVS after injections. **(A)** showed a transverse cross-sectional image at transverse process, a typical ultrasound image during TPVB using TI approach. **(B)** showed a sagittal cross-sectional image at the thoracic PVS, a typical ultrasound image during TPVB using SI approach. **(C)** PVS of T6-7 segment was stained using SI approach. **(D)** PVS of T6-7 to T3-4 segments were stained using TI approach. Yellow arrowheads indicated injection needle. Upward white triangles indicated pleura. ^*^ indicated the injection site. TP, transverse process; PVS, paravertebral space; TPVB, thoracic paravertebral block; TI, transverse *in-plane*; SI, sagittal *in-plane*.

Paravertebral injections were performed at T6-7 level using ultrasound guidance techniques based on established methods (17). 21-gange nerve blockade needle (HAKKO, Hakko co., Ltd., Japan) was used for all TPVB. Briefly, for TI approach (18), the linear transducer (13-6 MHz) was positioned transversely at T6. The corresponding transverse process, spinous process, internal intercostal membrane, and pleura were visualized via ultrasound. The transverse process reflection served as an anatomical guide for locating the injection site. Using an *in-plane* approach, the needle was advanced from lateral to medial under real-time ultrasound visualization. The needle tip was then carefully directed to the triangular area delineated by the parietal pleura, internal intercostal membrane, and intercostal muscles (Figure 1). 20 ml of 0.02% methylene blue contrast dye (diluted by normal saline) was injected. Correct needle placement was confirmed by anterior displacement of the parietal pleura under real-time ultrasound guidance. For the SI approach (19), the transducer was placed lateral to the midline in a sagittal orientation. The midpoint of the transducer was aligned between two adjacent transverse processes. The needle was inserted *in-plane* at the lower border of the transducer and advanced in a straight sagittal direction, cephalad and anteriorly, toward the PVS (Figure 1). Correct needle placement was confirmed by anterior displacement of the parietal pleura during injection. Following bilateral injections, the cadaver was repositioned from prone to supine.

### 2.2 Anatomical dissection

All anatomical dissections were carried out with the cadavers placed in the supine position. The anterior thoracic wall and the thoracic contents were removed. Subsequently, the posterior thoracic wall and associated paravertebral and intercostal spaces from T1 to T12 was exposed, permitting a clear observation. Cadaveric dissection was initiated directly following the injection and performed by a dedicated anatomist assisted by two trained technicians.

### 2.3 Outcome measures

The primary outcome was the number of PVS exhibiting contrast dye staining, compared between the two ultrasound-guided approaches. Secondary outcome included (1) success rate of TPVB for each approach, defined as the presence of dye in at least one PVS, confirmed by anatomical dissection. (2) the spread distances and total area of dye along the intercostal spaces for each approach. The sympathetic chain lies anterior to the rib head, and therefore, the origin point for the intercostal spread distance measurement was the sympathetic chain. The endpoint was the furthest point of dye diffusion along the rib. The total intercostal spread area was calculated as the sum of the spread area for each individual intercostal space. To calculate the area of a single intercostal space, we approximated the intercostal space as a rectangle, multiplying the diffusion distance by the width of the intercostal space to obtain the area of spread within that specific intercostal space. (3) incidence of thoracic sympathetic chain staining for each approach.

### 2.4 Statistical analysis

Continuous variables are presented as mean ± standard deviation or median [interquartile range (IQR)], as appropriate based on distribution. Categorical variables are expressed as numbers and percentages. The related-samples Wilcoxon signed rank test was used to assess differences between two ultrasound-guided approaches in the number of paravertebral segments stained, the intercostal spread distance and area, and the cephalad vs. the caudal dye spread. The McNemar test was used to compare the probability of sympathetic chains staining between two approaches. The above statistical analyses were performed with IBM SPSS statistics 29.0 software (IBM, Armonk, NY, USA). A generalized estimating equation (GEE) model was used (1) to estimate the association between the odds of certain PVS segment staining and injection approach adjusting for certain paravertebral segment; (2) to estimate the association between the continuous distance staining of certain paravertebral segment intercostal space and injection approach adjusting for certain paravertebral segment; (3) to estimate the association between the odds of thoracic sympathetic chain staining and the number of PVS stained. We firstly fitted GEE models with an interaction term between injection approach and segment; if the interaction term was not significant, it was further removed from the model and results were retrieved from models only including main effects. Cluster effect was set among observations within the same cadaver. Correlation structure was set as independence in all the GEE models. GEE model was conducted using the “geepack” package version 1.3.12 and R version 4.4.2. Statistical significance was defined as a two-sided *p* value of less than 0.05.

## 3 Results

A total of 5 fresh un-embalmed human cadavers (two females and three males) were included in the study. The mean age at death was 85.5 yr (range 82–101). Each cadaver received bilateral paravertebral injections at T6-7, with the TI approach performed on the left side and the SI approach on the right. The PVS were successfully identified and reached in all cadavers. Contrast dye was present in PVS following all injections. An illustrative example of contrast dye spread is shown in Figures 1C, D.

### 3.1 Paravertebral spread

**Figure 2:**
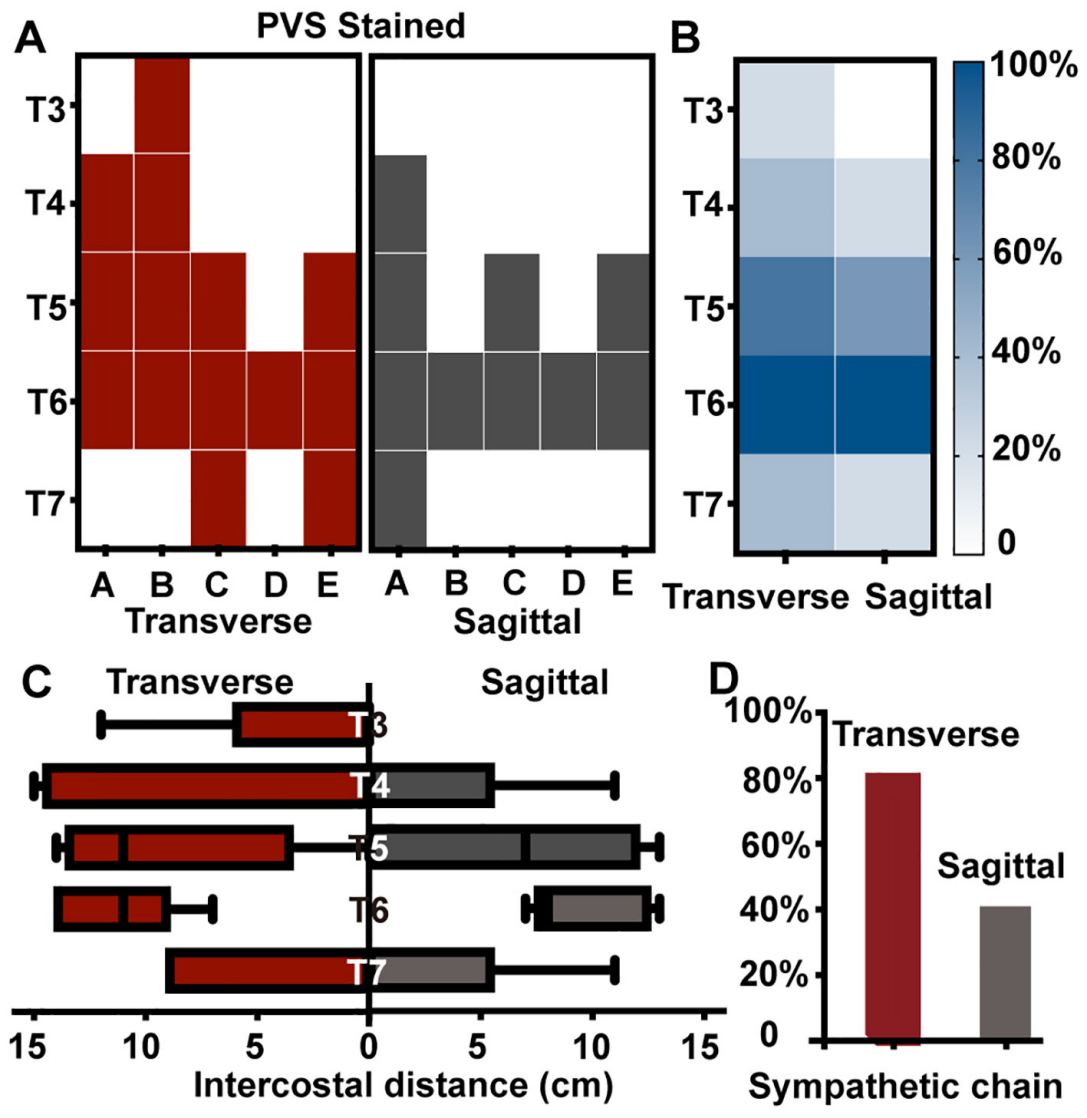
Contrast dye spread after ultrasound-guided paravertebral injections at T6-7. **(A)** showed the contrast dye distribution in PVS of 10 injections on 5 cadavers. A-E indicated the five cadavers. PVS stained after transverse TI approach were colored in red, while after SI approach were in black. **(B)** presented a heatmap summarizing the contrast dye distribution in all five cadavers. The intensity of the color corresponded to the probability of staining at each segment. Darker colors indicated a higher probability of staining. **(C)** showed the distance of each segment after ultrasound-guided paravertebral injections. Box plot showed the median, interquartile range, minimum and maximum values. **(D)** showed the percentage of sympathetic chain stained after paravertebral injections. Gray bars presented the results of SI approach, and red bars presented the results of TI approach. PVS, paravertebral space; TI, transverse *in-plane*; SI, sagittal *in-plane*.

The extent of paravertebral contrast dye spread was variable. Anatomical dissection revealed a median dye spread of 3 segments (3, 3) in the TI approach group and 2 segments (1, 2) in the SI approach group. There was no statistically significant difference in the number of PVS segments stained between the two approaches (*p* = 0.26). Figures 2A, B displayed the dye spread for all injections. GEE analyses did not demonstrate a statistically significant association between the odds of dye spread in specific PVS and injection approach (SI vs. TI) (Table 1). Regardless of the injection approach, a significant higher odds of staining was observed in PVS of T5-6 (OR, 13.19; 95% CI, 2.83 to 61.41) and T6-7 (OR, 2.01 × 10^19^; 95% CI, 6.77 × 10^18^ to 5.97 × 10^19^).

**Table 1:** Association between the odds of PVS stained/continuous intercostal spread distance and the injection approach, adjusting for vertebral segment using GEE models^**^.

**Segment**	**Binary PVS stained outcome**		**Continuous intercostal spread distance**	
	**OR**†**(95% CI)**	***P*** **value**	**Mean difference** ‡ **(95% CI)**	***P*** **value**
**T3-4**				
TI vs. SI approach	1.53 (0.81 to 2.87)	0.19	1.00 (−0.03 to 0.16)	0.13
T3-4 vs. other segments	0.42 (0.05 to 3.26)	0.41	−1.07 (−3.42 to 1.27)	0.37
**T4-5**				
TI vs. SI approach	1.53 (0.81 to 2.88)	0.19	1.00 (−0.30 to 2.30)	0.13
T4-5 vs. other segments	1.83 (0.36 to 9.18)	0.47	1.98 (−2.34 to 6.31)	0.37
**T5-6**				
TI vs. SI approach	1.63 (0.78 to 3.42)	0.19	1.00 (−0.30 to 2.30)	0.13
T5-6 vs. other segments	13.19 (2.83 to 61.41)	0.001	5.91 (2.68 to 9.14)	< 0.01
**T6-7**				
TI vs. SI approach	1.96 (0.65 to 5.89)	0.23	1.00 (−0.30 to 2.30)	0.13
T6-7 vs. other segments	2.01 × 10^19^ (6.77 × 10^18^ to 5.97 × 10^19^)	< 0.001	9.07 (7.45 to 10.70)	< 0.01
**T7-8**				
TI vs. SI approach	1.53 (0.81 to 2.88)	0.19	1.00 (−0.30 to 2.30)	0.13
T7-8 vs. other segments	1.83 (0.72 to 4.62)	0.20	0.78 (−1.06 to 2.63)	0.41

**The binary PVS stained outcome was regressed against binary injection approach (TI vs. SI) with logit link function (GEE logistic regression) in which odds ratio was reported as effect size; the continuous intercostal spread distance was regressed against binary injection approach (TI vs. SI) with identity link function (GEE linear regression). In both models, a covariate of “segment” was included to compare the outcome at a specific paravertebral segment (e.g., T3-4) vs. the average outcome across all other twelve levels. There were no significant interaction effects from all the GEE models.

†Represented the strength of association between the binary PVS stained outcome and the explanatory variables (injection approach and specific paravertebral segment). For example, 1.53, the first OR reported in the table, meant the odds of PVS staining at T3-4 (a measure of probability for PVS stained) was 53% higher with TI approach than with the SI approach. An OR greater than 1 signified the probability of PVS stained was higher with the TI approach compared with SI approach. Similarly, an OR of 0.42 for T3-4 vs. other segments indicated that the odds of PVS staining at the T3-4 segment was 58% lower than at other segments. An OR less than 1 signified the probability of PVS staining was lower in the T3-4 segment compared with other segments.

‡Represented the estimated average difference in intercostal spread distance between the TI group and SI group. For example, 1.00 cm, the first mean difference reported in the table, indicated the intercostal spread distance at T3-4 level after injection with TI approach was on average 1.00 cm longer than that with SI approach. A positive mean difference signified a longer intercostal spread distance with TI approach compared with SI approach. Similarly, mean difference of−1.07 cm for T3-4 vs. other segments signified that the intercostal spread distance was shorter at T3-4 segment than that in other segments. A negative mean difference signified a shorter intercostal spread distance at T3-4 segment compared to other segments. GEE, generalized estimating equations; OR, Odds ratio; PVS, paravertebral space; TI, transverse in-plane; SI, sagittal in-plane; CI, confidence interval.

Comparison of cephalad vs. caudal dye spread in PVS relative to the T6-7 puncture level revealed a cephalad distribution of 1 (0, 2) vertebral segment and a caudal distribution of 0 (0, 1) segment. The dye distributed significantly more in a cephalad direction compared to a caudal direction (*p* = 0.04).

### 3.2 Intercostal spread

The distances and area of dye spread within the intercostal spaces for two approaches were compared. Although the TI approach appeared to result in greater intercostal spread than the SI approach, the difference was not statistically significant [for distance, see Figure 2C; for area, TI vs. SI = 55.1 (30.1, 76.0) cm^2^ vs. 38.3 (7.8, 82.6) cm^2^, *p* = 0.50]. GEE analysis was conducted to assess the correlation between dye spread distance within specific intercostal spaces and injection approach adjusting for thoracic segment. These analyses did not reveal a statistically significant correlation (Table 1). However, a significantly longer spread distance was revealed in T5-6 and T6-7 intercostal space regardless of the injection approach (Table 1).

### 3.3 Thoracic sympathetic chain involvement

The spread of contrast dye to the thoracic sympathetic chain was also investigated. Dye was observed surrounding the sympathetic chain in 4 (80%) cadavers using TI approach and 2 (40%) cadavers using SI approach. There was no significant difference in the probability of sympathetic chain stained between two approaches (*p* = 0.50, McNemar test, Figure 2D). GEE analyses demonstrated a strong positive association between the number of PVS segments stained and the probability of sympathetic chain staining (*p* = 0.003, OR, 6.71; 95% CI, 1.88 to 23.93).

## 4 Discussion

This cadaveric study aimed to compare the spread pattern of ultrasound-guided TPVB at T6-7 using 20 mL of 0.02% methylene blue via TI and SI approaches. While the TI approach resulted in a mean cranio-caudal spread encompassed 3 (3,3) vertebral levels, 55.1 (30.1, 76.0) cm^2^ of intercostal spaces, and 80% of sympathetic chain staining, compared to 2 (1,2) vertebral levels, 38.3 (7.8, 82.6) cm^2^, and 40% with the SI approach, no statistical differences were found. Importantly, regardless of the approach, dye distribution was predominantly cephalad, and a reliably staining of PVS and intercostal space at the injection segment and the immediately adjacent cephalad segment was indicated. Furthermore, we observed a positive correlation between the extent of paravertebral spread and the likelihood of sympathetic chain staining.

The 100% success rate in achieving PVB with two different approaches underscore the feasibility and reliability of ultrasound-guided TPVB. Previous studies have reported variable vertebral level staining after ultrasound-guided PVB with sagittal *in-plane* approach (2-6 levels) and the transverse *in-plane* approach (2-10 levels) (20, 21). Ruscio et al. also compared contrast dye spread between the transverse and sagittal approaches, reporting mean vertebral level staining of 3 (range 1-5) and 2 (range 1-4), respectively (22). In conjunction with our findings, these results suggested that the TI approach may result in a slightly more segments of vertebral levels stained compared to the SI approach. Although volunteer studies indicated that the spread of LA in the PVS on MRI scan did not perfectly match the area of sensory block (23), the distribution of injectate within the PVS provided an important anatomical basis for understanding the mechanism of TPVB. More research is warranted to investigate the relationship between LA spread in the PVS and the distribution of sensory blockade in patients.

We found that PVS of injection segment and the immediately adjacent cephalad segment were consistently and reliably stained by contrast dye regardless of injection approach. In addition, the dye distributed significantly more in a cephalad segment compared to a caudal segment. Those results indicated a predominantly cephalad spread of the injectate, irrespective of the approach. This predominant cephalad spread may be attributed to the anatomical constraints imposed by the superior costotransverse ligaments (SCTL), which form the posterior border of the PVS. The SCTL originates from the superior border of the rib neck and extends supero-laterally to attach to the inferior border of the transverse process of the vertebra immediately above (8, 24). This anatomical arrangement results in a larger cephalad space within the PVS compared to the caudal space at the same segmental level, potentially facilitating preferential cephalad injectate diffusion. However, a study by Marhofer et al. (23) in volunteers using ultrasound-guided transverse *out-of-plane* TPVB technique, with MRI scanning after injection, found a predominantly caudal distribution of LA. We acknowledged that the *out-of-plane* technique and the use of living volunteers in that study was different from our study. Employing the *out-of-plane* technique, the needle tip visualization is poor, which could potentially result in a different puncture location. Furthermore, the cadaveric model intrinsically differs from the living patient. These differences include variations in compartmental and tissue pressures, as well as the absence of dynamic uptake of injected solutions. These factors, in aggregate, may contribute to discrepancies between two studies. Nevertheless, it also suggests that variations in technique can influence the pattern of injectate spread. Further studies are needed to validate these observations. Given the observed cephalad spread in our study, selecting a TPVB injection site caudal to the intercostal nerve innervating the surgical incision may be a prudent clinical strategy.

In the present study, we observed the diffusion of dye into the space between the parietal pleura and the posterolateral chest wall following ultrasound-guided TPVB. While with ultrasound guidance the correct needle placement of PVB was confirmed by anterior displacement of the parietal pleura. Consequently, the diffusion of dye beneath the parietal pleura indicates a successful PVB. This observation is consistent with findings reported in previous publications (21, 25–27). The intercostal nerves, after exiting the intervertebral foramen, travel inferior to the pleura before they travel within the plane between the internal intercostal muscle and the innermost intercostal muscle. Therefore, after ultrasound-guided TPVB, LA diffusion beneath the pleura effectively blocks the proximal segment of the intercostal nerve. In this study, the distance and area of intercostal spread was used as an indirect measure of the volume of LA pooling around the intercostal nerve. It is generally accepted that a larger volume of pooled LA correlates with a longer duration of action.

In our study, there were no instances where intercostal spread occurred in the absence of PVS spread. Specifically, when the PVS was stained, the intercostal space was also stained; conversely, when the PVS was not stained, neither was the intercostal space. However, we did observe that intercostal spread sometimes occurred via diffusion from an adjacent intercostal space, rather than directly from the PVB injection in the same intercostal level. This suggested that LA diffusion beneath the parietal pleura may be one of the mechanisms contributing to intercostal nerve blockade, although the direct diffusion from corresponding PVS plays a crucial role.

We also observed a high incidence of sympathetic chain staining, averaging 60% (80% with the TI approach and 40% with the SI approach). This finding was consistent with prior research (21, 28). Furthermore, our study suggested that a more extensive dye spread in PVS correlated with a higher odd of sympathetic chain staining, regardless of the injection approach. While sympathetic blockade may contribute to visceral pain control compared to peripheral intercostal nerve block, it can also lead to side effects such as hypotension and bradycardia (29). Therefore, clinicians should be cognizant of the potential for sympathetic blockade following TPVB and be prepared to manage associated side effects.

## 5 Limitations

There were several limitations in the present study. First, the small sample size in this cadaveric study may have limited our statistical power to detect subtle, yet potentially clinically relevant differences. The scarcity of donated cadaveric specimens presented a significant challenge for larger sample size. Second, the cadaver model inherently differs from the living patient due to variations in compartmental and tissue pressures, and the absence of dynamic uptake of injections. These factors may limit the extent and pattern of injectate distribution compared to living human subjects. The injectate spread observed in cadavers may not fully replicate the situation in living patients. Thus, further clinical studies that enroll volunteers or patients are still required to evaluate the similarities and differences between the two injection approaches with respect to clinical efficacy. We noticed that positioning may influence the spread of injectate after TPVB in living patients (30). In the prone position, the injectate could even reach the anterior thorax. Owing to the different factors between cadaver model and living patient, our study presented the spread pattern without further redistribution. It is also worth noting that previous publications investigating injectate distribution after TPVB with cadaveric model have similarly utilized a prone position (21, 22). Next, we were unable to assess the potential spread of the dye to the epidural space due to experimental limitation. In clinical practice TPVB is exclusively performed for unilateral analgesia purpose. We believed that the distribution pattern on the blocked side is of primary concern. Finally, methylene blue was used as contrast dye because it is inexpensive, universally obtainable, almost non-toxic, and provides high-contrast visualization, which was frequently used in anatomic studies (26, 31, 32). We acknowledged that anatomic dissection may cause mechanical displacement of dye and could overestimate the true spread—a limitation common to all cadaveric staining investigations using anatomic dissection technique. Nevertheless, direct anatomical exposure remains an important method for defining detailed perineural and fascial distribution. Future work using CT or MRI with iodinated or gadolinium-based contrast could corroborate our findings and quantify spread *in vivo*, although such approaches may not resolve perineural and fascial anatomical details. Despite these limitations, our study provided novel evidence contributing to a better understanding of the differences in spread patterns between two distinct ultrasound-guided TPVB techniques.

## 6 Conclusion

This cadaveric study demonstrated the ability to reliably target the thoracic PVS with ultrasound guidance and a predominantly cephalad spread of the injectate, irrespective of the injection approach (TI or SI). Meanwhile, a more segments of PVS stained, a higher possibility of sympathetic chain stained. While the TI approach tended to result in a slightly greater number of stained PVS segments, a longer intercostal spread distance, and a higher incidence of sympathetic chain staining compared to the SI approach, these differences were not statistically significant. These anatomical findings may contribute to optimizing clinical practice and potentially improving patient outcomes.

## Data Availability

The raw data supporting the conclusions of this article will be made available by the authors, without undue reservation.
